# Glucose- and glutamine-driven de novo nucleotide synthesis facilitates WSSV replication in shrimp

**DOI:** 10.1186/s12964-025-02186-z

**Published:** 2025-04-22

**Authors:** Cong-Yan Chen, Chih-Ling Chen, Yen Siong Ng, Der-Yen Lee, Shih-Shun Lin, Chien-Kang Huang, Ramya Kumar, Han-Ching Wang

**Affiliations:** 1 Department of Biotechnology and Bioindustry Sciences, National Cheng Kung University, Tainan, Taiwan; 2 International Center for the Scientific Development of Shrimp Aquaculture, National Cheng Kung University, Tainan, Taiwan; 3 Graduate Institute of Integrated Medicine, China Medical University, Taichung, Taiwan; 4 Institute of Biotechnology, National Taiwan University, Taipei, Taiwan; 5 Department of Engineering Science and Ocean Engineering, National Taiwan University, Taipei, Taiwan

**Keywords:** White shrimp, White spot syndrome virus, Warburg effect, Pentose phosphate pathway, de novo nucleotide metabolism, in vivo stable-isotope tracing metabolomics

## Abstract

**Background:**

Viruses rely on host metabolism to complete their replication cycle. White spot syndrome virus (WSSV), a major pathogen in shrimp aquaculture, hijacks host metabolic pathways to fulfill its biosynthetic and energetic needs. Previous studies have demonstrated that WSSV promotes aerobic glycolysis (Warburg effect) and glutaminolysis during its replication stage (12 hpi). Therefore, glucose and glutamine serve as crucial metabolites for viral replication. Additionally, de novo nucleotide synthesis, including the pentose phosphate pathway and purine/pyrimidine synthesis, is significantly activated during WSSV infection. However, the precise association between WSSV and host glucose and glutamine metabolism in driving de novo nucleotide synthesis remains unclear. This study aimed to investigate the involvement of glucose and glutamine in nucleotide metabolism during WSSV replication and to elucidate how WSSV reprograms these pathways to facilitate its pathogenesis.

**Methods:**

To assess changes in metabolic flux during WSSV replication, LC-ESI-MS-based isotopically labeled glucose ([U-^13^C] glucose) and glutamine ([A-^15^N] glutamine) were used as metabolic tracers in in vivo experiments with white shrimp (*Litopenaeus vannamei*). The in vivo experiments were also conducted to measure the expression and enzymatic activity of genes involved in nucleotide metabolism. Additionally, in vivo dsRNA-mediated gene silencing was employed to evaluate the roles of these genes in WSSV replication. Pharmacological inhibitors targeting the Ras-PI3K-Akt-mTOR pathway were also applied to investigate its regulatory role in WSSV-induced nucleotide metabolic reprogramming.

**Results:**

The metabolite tracking analysis confirmed that de novo nucleotide synthesis was significantly activated at the WSSV replication stage (12 hpi). Glucose metabolism is preferentially reprogrammed to support purine synthesis, while glutamine uptake is significantly increased and contributes to both purine and pyrimidine synthesis. Consistently, gene expression and enzymatic activity analyses, along with gene silencing experiments, indicated the critical role of de novo nucleotide synthesis in supporting viral replication. However, while the inhibition of the Ras-PI3K-Akt-mTOR pathway suggested its involvement in regulating nucleotide metabolism, no consistent effect on WSSV replication was observed, suggesting the presence of alternative regulatory mechanisms.

**Conclusion:**

This study demonstrates that WSSV infection induces specific metabolic reprogramming of glucose and glutamine utilization to facilitate de novo nucleotide synthesis in shrimp. These metabolic changes provide the necessary precursors for nucleotide synthesis, supporting WSSV replication and pathogenesis. The findings offer novel insights into the metabolic strategies employed by WSSV and suggest potential targets for controlling WSSV outbreaks in shrimp aquaculture.

**Supplementary Information:**

The online version contains supplementary material available at 10.1186/s12964-025-02186-z.

## Background

Shrimp aquaculture is the commercial production of shrimp species, with millions of tonnes of shrimp products traded globally each year. Since the 1950s, there has been an increasing demand for shrimp as the human population skyrocketed [1], but at the same time, recurrent outbreaks of pathogen-caused disease have led to enormous economic losses in the shrimp industry [2, 3]. In the case of viral diseases, the host metabolic system is typically hijacked and rerouted to create an appropriate cellular environment with an abundance of the metabolites that are required by most viruses to support pathogenesis [4, 5]. In virally induced host cell reprogramming, a number of metabolic pathways, including nucleotide synthesis, glutaminolysis, and fatty acid synthesis, are promoted during virus infection [6, 7]. Elucidating pathogen-host interactions and pathogen-induced host metabolic alterations are therefore fundamental to our understanding of these diseases.

White spot disease (WSD) is caused by the white spot syndrome virus (WSSV), a large DNA virus first identified in China in 1992. It can cause 90–100% mortality within a week, with symptoms that include reduced food consumption, calcium accumulation on the exoskeleton (white spots), and red discoloration of the body [8–11]. Previous reports have indicated that in infected cells, WSSV induces the Warburg effect, with its hallmark characteristics of a high rate of glucose oxidation and the accumulation of extracellular lactate from the lactic fermentation pathway even in the presence of oxygen [12]. Until recently, the virus-induced Warburg effect had only been reported in vertebrate pathogen infections, and WSSV was the first virus shown to induce this effect in invertebrates [13]. It was subsequently found that WSSV induces metabolic reprogramming in glycolysis, TCA cycle, glutaminolysis, and amino acid metabolism at the WSSV genome replication stage (12 h post WSSV injection, 12 hpi), and in lipid metabolism at the late stage of WSSV replication (24 h post WSSV infection, 24 hpi) [14–18]. However, to date, there has been no attempt to comprehensively investigate and analyze the changes in nucleotide metabolism during WSSV infection.

Viral infections typically usurp and facilitate host de novo nucleotide synthesis to address the virus’s requirements for rapid genome replication [19]. de novo nucleotide synthesis involves glycolysis, the pentose phosphate pathway (PPP), as well as purine and pyrimidine synthesis. This process requires two essential upstream inputs: glucose and glutamine. In addition to its role in ribose synthesis, the PPP also serves as the primary mechanism for generating NADPH, which is required for various cellular processes, including DNA synthesis, amino acid synthesis, fatty acid synthesis, and the reduction of oxidative stress [20–23]. Purine synthesis, which occurs downstream of the PPP, not only produces the basic building blocks for DNA and RNA synthesis, it also provides energy and several factors (e.g. cAMP, NADH, and coenzyme A) that are important for the maintenance of biological processes. We note that activation of purine synthesis has been observed in cancer cells and virus-infected cells [24, 25], and that purine derivatives continue to be explored as therapeutic agents for cancer and virus infection [26, 27]. Lastly, the importance of pyrimidine synthesis, the other metabolic branch that catalyzes ribose to generate nucleotides, is suggested by the fact that several genes related to this pathway, including ribonucleotide reductase (RR), thymidylate synthase (TS), thymidine kinase (TK) and thymidylate kinase (TMK) are encoded on the WSSV genome [28–30].

Taken together, the above reports all suggest that de novo nucleotide synthesis is likely to be activated during WSSV infection. However, despite its evident importance, the means by which WSSV regulates the production of host nucleotides is not well understood, and the involvement of glucose/glutamine metabolism also remains unclear. In vertebrates, multiple studies related to cancer progression and viral infections have shown a significant increase in the mRNA expression of genes involved in de novo nucleotide synthesis, and have further shown that suppression of these specific genes significantly decreased tumour proliferation and viral replication [31–34]. In the present study, using bioinformatic analysis, we first verified the strong association between genes involved in de novo nucleotide synthesis and WSSV-affected differentially expressed genes (DEGs) during WSSV infection. Next, we conducted in vivo animal experiments using labelled [U-^13^C]glucose and [A-^15^N]glutamine to assess the flux of glucose- and glutamine-derived metabolites during WSSV infection. Lastly, double stranded RNA (dsRNA) mediated gene silencing was used to explore the role of de novo nucleotide synthesis in WSSV pathogenesis and investigate the underlying modulatory mechanisms, while pharmacological inhibitors were used to investigate the possible regulatory role of the Ras-PI3K-Akt-mTOR pathway.

## Methods

### Experimental animals and WSSV inoculum

White shrimp (*Litopenaeus vannamei*, 3–5 g body weight) were obtained from the Department of Aquaculture, National Pingtung University of Science and Technology (NPUST) and used for in vivo animal experiments. The WSSV (Taiwan isolate, GenBank accession no. AF440570) stock was prepared from hemolymph of WSSV-infected moribund SPF (specific pathogen free) shrimp. The viral inoculum was prepared from the stock for intramuscular injection by dilution (10^− 4^) in 1x phosphate buffer saline (PBS) (137 mM NaCl, 2.7 mM KCl, 10 mM Na_2_HPO_4_, and 2 mM KH_2_PO_4_). The WSSV challenge dosage (100 µL/3 g shrimp) resulted in a 50% cumulative mortality at 72 h post WSSV injection, while the control group were given 1x PBS (100 µL/3 g shrimp) as described in Su et al. (2014) and Ng et al. (2022) [13, 14]. Before the experiments, shrimp were maintained for 1∼2 days in sterilized seawater (20 parts-per-thousand) at 27 °C. At 12 and 24 h post WSSV challenge, hemocyte samples were collected and used for metabolomic analysis, enzyme activity assays, and the synthesis of complementary DNA to measure the expression of host and viral genes. In addition, pleopod tissues were collected and used to measure WSSV genomic DNA copies.

### Gene-to-gene correlation network analysis

An in-house *L. vannamei (Litopenaeus vannamei)* stomach transcriptomic database, as described previously [35], was established using next generation sequencing and applied to ContigViews (http://www.contigviews.bioagri.ntu.edu.tw/). To create a gene-to-gene correlation network, we first identified differentially expressed genes using a criterion of at least a 2-fold change between the PBS-treated group and the WSSV-inoculated group at 1, 6, 12, and 24 h post injection, respectively. A Pearson correlation coefficient threshold as|r| ≥ 0.6. was applied to build a network that included only strongly correlated contigs. Critical genes involved in de novo nucleotide synthesis were then identified according to the annotations of their respective contigs, and the remaining contigs in the network were then subjected to KEGG pathway analysis via DAVID (https://david.ncifcrf.gov/).

### Cloning of partial cDNA sequences of *LvG6PDH*, *LvTKT*, *LvPRPS*, *LvATase*, *LvADSS*, *LvIMPDH*, *LvCAD*, *LvDHODH*, *LvUMPS*, and *LvCMPK*

The same in-house *L. vannamei* stomach transcriptomic database was used in combination with the NCBI (National Center for Biotechnology Information) BLAST function to identify target genes. Contigs PVHP204597.4, PVHP71465.2, PVHP205382.2, PVHP244493.1/PVHP244501.1, PVHP165389.1, PVHP227689.1, PVHP274591.1, PVHP148100.1, PVHP164355.1, and PVHP117215.1 were found to match *L. vannamei G6PDH* (glucose-6-phosphate dehydrogenase), *TKT* (transketolase), *PRPS* (phosphoribosyl pyrophosphate synthetase), *ATase* (amidophosphoribosyltransferase), *ADSS* (adenylosuccinate synthetase), *IMPDH* (inosine monophosphate dehydrogenase), *CAD* (carbamoyl-phosphate synthetase 2, aspartate transcarbamylase, and dihydroorotase), *DHODH* (dihydroorotate dehydrogenase), *UMPS* (uridine monophosphate synthase), and *CMPK* (cytidine/uridine monophosphate kinase), respectively. Primer sets were designed based on these contigs to amplify partial sequences of the corresponding genes (Table 1).

**Table 1:** Primer sets used in the present study

Gene	Primer	Primer sequence (5’-3’)	Function
*G6PDH* (PVHP204597.4)	*G6PDH*-qF	5’-CGCCACCATCAACAACGA-3’	Real-time PCR
	*G6PDH*-qR	5’-AGGGCCTTTCCGCATCTG-3’	Real-time PCR
	*G6PDH*-dsF	5’-AAGCTGTCGAACCACCTG-3’	Cloning
*TKT* (PVHP71465.2)	*TKT*-qF	5’-CACTGCCTTCCAGCATGACA-3’	Real-time PCR
	*TKT*-qR	5’-ATTCCAGCCAAAACCTTCGA-3’	Real-time PCR
	*TKT*-dsR	5’-GAGACAAAAGCAACAGTACGGT-3’	Cloning
	*TKT*-qF-T7	5’-*TAATACGACTCACTATAGGGAGA*CACTGCCTTCCAGCATGACA-3’	dsRNA synthesis
	*TKT*-dsR-T7	5’-*TAATACGACTCACTATAGGGAGA*GAGACAAAAGCAACAGTACGGT-3’	dsRNA synthesis
*PRPS* (PVHP205382.2)	*PRPS*-qF	5’-TCTATCGCGAGCCCCAATT-3’	Real-time PCR
	*PRPS*-qR	5’-GGCCACGGAGAGCATGTT-3’	Real-time PCR
	*PRPS*-dsR	5’-AGACTCCTTCATGTGCTG-3’	Cloning
	*PRPS*-qF-T7	5’-*TAATACGACTCACTATAGGGAGA*TCTATCGCGAGCCCCAATT-3’	dsRNA synthesis
	*PRPS*-dsR-T7	5’-*TAATACGACTCACTATAGGGAGA*AGACTCCTTCATGTGCTG-3’	dsRNA synthesis
*ATase* (PVHP244493.1, PVHP244501.1)	*Atase*-qF	5’-TCCGTTGATGCTGATATTATTTCC-3’	Real-time PCR
	*Atase*-qR	5’-ATCCCATAGCAGCCGGAGTA-3’	Real-time PCR
*ADSS* (PVHP165389.1)	*ADSS*-qF	5’-CGGATGCGTCCCCTTGT-3’	Real-time PCR
	*ADSS*-qR	5’-GTTGCTATGGAGACACTTGTGGAT-3’	Real-time PCR
*IMPDH* (PVHP227689.1)	*IMPDH*-qF	5’-AAGCACAGGAGGTCATGAAAGTC-3’	Real-time PCR
	*IMPDH*-qR	5’-GGCAAACAGGGTCACGAATG-3’	Real-time PCR
	*IMPDH*-dsR	5’-TCAACACCAGCATTGACAAG-3’	Cloning
	*IMPDH*-qF-T7	5’-*TAATACGACTCACTATAGGGAGA*AAGCACAGGAGGTCATGAAAGTC-3’	dsRNA synthesis
	*IMPDH*-dsR-T7	5’-*TAATACGACTCACTATAGGGAGA*TCAACACCAGCATTGACAAG-3’	dsRNA synthesis
*CAD* (PVHP274591.1)	*CAD*-qF	5’-CGGTTCAAGTTTTCGCGTATG-3’	Real-time PCR
	*CAD*-qR-F2-dsR	5’-TGGGCTGACTTGAGGTTGGT-3’	Real-time PCR
	*CAD*-F1-dsF	5’-CATTGGTCCTCCGTCAAT-3’	Cloning
	*CAD*-F1-dsR	5’-CCGTGATTCTGAGACGTG-3’	Cloning
	*CAD*-F2-dsF	5’-AGAAGCACAACATCATGCCT-3’	Cloning
	*CAD*-F3-dsF	5’-AACATGTCCTGAGTGTTC-3’	Cloning
	*CAD*-F3-dsR	5’-GCATACTGTGTGACATAG-3’	Cloning
	*CAD*-F1-dsF-T7	5’-*TAATACGACTCACTATAGGGAGA*CATTGGTCCTCCGTCAAT-3’	dsRNA synthesis
	*CAD*-F1-dsR-T7	5’*-TAATACGACTCACTATAGGGAGA*CCGTGATTCTGAGACGTG-3’	dsRNA synthesis
	*CAD*-F2-dsF-T7	5’-*TAATACGACTCACTATAGGGAGA*AGAAGCACAACATCATGCCT-3’	dsRNA synthesis
	*CAD*-F2-dsR-T7	5’-*TAATACGACTCACTATAGGGAGA*TGGGCTGACTTGAGGTTGGT-3’	dsRNA synthesis
	*CAD*-F3-dsF-T7	5’-*TAATACGACTCACTATAGGGAGA*AACATGTCCTGAGTGTTC-3’	dsRNA synthesis
	*CAD*-F3-dsR-T7	5’-*TAATACGACTCACTATAGGGAGA*GCATACTGTGTGACATAG-3’	dsRNA synthesis
*DHODH* (PVHP148100.1)	*DHODH*-qF	5’-GGCAGCAGGATTTGATAAGCAT-3’	Real-time PCR
	*DHODH*-qR	5’-AAAGCTGAATCCCATACGAAACA-3’	Real-time PCR
	*DHODH*-dsR	5’-AGAAACGGTTGTGTTGCTT-3’	Cloning
	*DHODH*-qF-T7	5’-*TAATACGACTCACTATAGGGAGA*GGCAGCAGGATTTGATAAGCAT-3’	dsRNA synthesis
	*DHODH*-dsR-T7	5’-*TAATACGACTCACTATAGGGAGA*AGAAACGGTTGTGTTGCTT-3’	dsRNA synthesis
*UMPS* (PVHP164355.1)	*UMPS*-qF-dsF	5’-AACTCATGTGGGCCAAAC-3’	Real-time PCR
	*UMPS*-qR	5’-GGCTACCCCGCAAACCAG-3’	Real-time PCR
	*UMPS*-dsR	5’-TAACTCCGCAGAGGTGGT-3’	Cloning
	*UMPS*-dsF-T7	5’-*TAATACGACTCACTATAGGGAGA*AACTCATGTGGGCCAAAC-3’	dsRNA synthesis
	*UMPS*-dsR-T7	5’-*TAATACGACTCACTATAGGGAGA*TAACTCCGCAGAGGTGGT-3’	dsRNA synthesis
*CMPK* (PVHP117215.1)	*CMPK*-qF	5’-CCTTGTTAGAACGTGCCATGAA-3’	Real-time PCR
	*CMPK*-qR	5’-CCGAGGAAATCCATCAATGAG-3’	Real-time PCR
	*CMPK*-dsF	5’-CCAAGCTTATGTTTTTGAATCGAATT-3’	Cloning
	*CMPK*-dsR	5’-CCGGATCCACTGAACAGCTTTTCAAC-3’	Cloning
	*CMPK*-dsF-T7	5’-*TAATACGACTCACTATAGGGAGA*ATGTTTTTGAATCGAATT-3’	dsRNA synthesis
	*CMPK*-dsR-T7	5’-*TAATACGACTCACTATAGGGAGA*ACTGAACAGCTTTTCAAC-3’	dsRNA synthesis
Luciferase	*Luc*-F	5’-CTGAATACAAATCACAGAATC-3’	Cloning
	*Luc*-R	5’-GCGAGAATCTGACGCAGGCAGT-3’	Cloning
	*Luc*-F-T7	5’-*TAATACGACTCACTATAGGGAGA*CTGAATACAAATCACAGAATC-3’	dsRNA synthesis
	*Luc*-R-T7	5’-*TAATACGACTCACTATAGGGAGA*GCGAGAATCTGACGCAGGCAGT-3’	dsRNA synthesis
*EF-1α*	*EF-1α*-qF	5’-ACGTGTCCGTGAAGGATCTGAA-3’	Real-time PCR
	*EF-1α*-qR	5’-TCCTTGGCAGGGTCGTTCTT-3’	Real-time PCR
WSSV *IE1*	*IE1*-qF	5’-CCAGGCCCAGTGTCATACG-3’	Real-time PCR
	*IE1*-qR	5’-AGAAATCTCATCACATGTCAAATCAGA-3’	Real-time PCR
WSSV *VP28*	*VP28*-qF	5’-AGTTGGCACCTTTGTGTGTGGTA-3’	Real-time PCR
	*VP28*-qR	5’-TTTCCACCGGCGGTAGCT-3’	Real-time PCR
Others	Anchor-dTV	5’-GACCACGCGTATCGATGTCGACTTTTTTTTTTTTTTTTV-3’	Reverse transcription

The primer set for the genes involved in de novo nucleotide synthesis was designed using an in-house *L. vannamei* transcriptomic database. Accession numbers are given below in parentheses.

The added T7 promoter sequence is in italics.

### Measurement of host genes and WSSV genes by Real-Time PCR

Pooled hemocyte samples were collected from each group (4 pools with 3 shrimp each) and mixed with an equal volume of ice-cold anticoagulant (450 mM NaCl, 10 mM KCl, 10 mM EDTA, 10 mM Tris, pH 7.45). Total RNA was extracted using REzol C&T Reagent (PT-KP200CT, PROtech) and subjected to cDNA synthesis using SuperScript™ II Reverse Transcriptase (18064-014, Invitrogen) in conjunction with Anchor-dTv primer (Table 1). SYBR^®^ FAST ABI Prism^®^ 2X qPCR Master Mix (KK4603, KAPA) and the Real-time PCR detection system (Bio-Rad) were used to quantify mRNA expression. The primer sets used for detecting the individual gene expression levels in Real-time PCR are listed in Table 1. Data were normalized relative to the expression of the housekeeping gene Elongation factor 1 alpha (*EF-1α*) and quantified by the 2^−ΔCT^ method [36]. The resulting values were then subjected to statistical analysis as described below.

### Quantification of the WSSV genome copy number using the IQ Real^™^ WSSV quantitative system

To quantify WSSV genomic DNA copy numbers after WSSV infection, a DTAB/CTAB DNA extraction kit (GeneReach) was used to extract genomic DNA from pooled pleopod tissues (4 pools of 3 shrimp each) and the IQ REAL™ WSSV Quantitative System used to determine WSSV genome copies. The WSSV genome copies determined for each group were then subjected to statistical analysis as described below.

### Using stable isotope-labeled glucose/glutamine tracer and liquid chromatography electrospray ionization mass spectrometry (LC-ESI-MS) to monitor metabolites in the hemocytes of WSSV-infected shrimp

LC-ESI-MS was used to detect and measure stable isotope-labeled metabolites within the de novo nucleotide synthesis pathways. Glucose inputs were labeled using a stable carbon isotope [U-^13^C]glucose (CLM-1396-PK, Cambridge Isotope Laboratories), while glutamine inputs were labeled with a stable nitrogen isotope [A-^15^N]glutamine (NLM-557-PK, Cambridge Isotope Laboratories). The animal trial protocol was adapted from He et al. (2019) [17]. In brief, [U-^13^C]glucose (450 µg/g shrimp) or [A-^15^N]glutamine (1 mg/g shrimp) was prepared by dissolving it in filtered 1x PBS after which it was injected into the abdominal hemal sinus at 12 or 24 hpi. At 10–30 minutes after tracer injection, hemocyte samples (4 pools with 3 shrimp each) were collected. Based on previous studies, we selected collection times of 10 and 30 minutes post-injection, as these intervals were found to most effectively capture metabolite flux [14, 17]. MeOH was used to extract the metabolites, which were then lyophilized and prepared for LC-ESI-MS analysis as follows: First, lyophilized samples were dissolved in 35 µL of ultrapure water, 5 µL of 0.3 M aniline/HCl (molar ratio: 5/1), and 5 µL of 20 mg/mL 1-Ethyl-3-(3-dimethylaminopropyl)-carbodiimide (EDC), followed by a 2-hour incubation. Next, 5 µL of 10% ammonium hydroxide were added to stop the reaction, followed by a half-hour incubation. Finally, the mixture was centrifuged at 14,000 rpm for 10 minutes and the supernatant subjected to LC-ESI-MS analysis in negative ion mode. The LC-ESI-MS analysis was performed using an ultra-performance liquid chromatography (UPLC) system (ACQUITY UPLC I-Class, Waters) and an ESI/APCI source of 4 kDa quadrupole time-of-flight (TOF) mass spectrometer (Waters VION, Waters) as described in Lin et al. (2023) and Huang et al. (2019) [37, 38]. In brief, elution started from 99% mobile phase A (ultrapure water + 0.1% formic acid) and 99% mobile phase B (100% methanol + 0.1% formic acid), held at 1% B for 0.5 minute, raised to 90% B in 5.5 minutes, held at 90% B for 1 minute, and then lowered to 1% B in 1 minute. The column was equilibrated by pumping 1% B for 4 minutes. The LC-ESI-MS chromatogram was set in ESI + with the following conditions: capillary voltage of 2.5 kV, source temperature of 100 °C, desolvation temperature at 250 °C, cone gas maintained at 10 L/h, desolvation gas maintained at 600 L/h, and acquisition by MS^E^ mode with range of m/z 100–1000 and 0.5 second scan time. MS data were analyzed with UNIFI software (Waters) and compound intensity was calculated by integrated quantification of the corresponding peak. The detected ^13^C-or ^15^N-labeled metabolites were first normalized to their corresponding sample weight, and the resulting data were then subjected to statistical analysis as described below.

### Evaluation of *TKT* enzyme activity in shrimp hemocytes after WSSV challenge

Shrimp hemocytes were collected at 12 and 24 h post WSSV injection and evaluated with a Transketolase Activity Assay Kit (Fluorometric) (K2004, Biovision). Hemocytes samples were harvested and homogenized in 100 µL of ice-cold assay buffer and centrifuged at 10,000×g for 15 minutes at 4 °C to collect the lysate supernatant. A Bradford assay with Protein Assay Dye Reagent Concentrate (5000006, Bio-Rad) was used to quantify total protein concentration. Subsequently, 5 µg of hemocyte lysate was brought to a final volume of 50 µL with *TKT* assay buffer. The reaction was initiated at 37 °C with the addition of 42 µL *TKT* assay buffer, 2 µL *TKT* substrate mix, 2 µL *TKT* developer, 2 µL *TKT* probe, and 2 µL enzyme mix. In addition, G3P standards supplied with the kit were prepared by adding 44 µL *TKT* assay buffer, 2 µL *TKT* developer, 2 µL *TKT* probe, and 2 µL enzyme mix. *TKT* activity was measured at Ex/Em = 535/595 nm every 1.5 minutes for 60 minutes. After measurement, two time points were chosen within the linear portion of the curve (t1 & t2) for each sample and readings applied to the G3P standard curve to determine ΔM pmol of G3P formed. Sample *TKT* Specific Activity was calculated as follows: ΔM x D / (Δt x P) (pmol / (min x µg)) = µUnits/µg (ΔM = G3P conc from the Standard Curve (pmol); ∆t = t2-t1 (min); D = Sample dilution factor; P = Sample used (in µg).

### Evaluation of *PRPS* enzyme activity in shrimp hemocytes after WSSV challenge

*PRPS* enzyme activity in shrimp hemocytes was measured using a PRECICE^®^ PRPP Assay Kit (K0709-04-2, NovoCIB). Hemocyte samples were homogenized in 100 µL of ice-cold assay buffer, and the homogenates were then centrifuged at 10,000×g for 15 minutes at 4 °C. Total protein concentrations in the supernatant were determined by a Bradford assay, after which 10 µg of the hemocyte lysate proteins was adjusted to a final volume of 10 µL with assay buffer and mixed with 200 µL reaction mix containing 190 µL assay buffer, 2 µL Cystine, 2 µL NAD, 2 µL ATP, and 4 µL *IMPDH*-HGPRT enzymes in two individual wells. After incubation at 37 °C for 15 minutes, the reaction was initiated by adding 2 µL P-ribose to one of two wells. *PRPS* activity was measured at 340 nm every 2 minutes for 60 minutes. After measurement, *PRPS* activity was calculated as follows: (Mean ARP5R- Mean ARblank)/(4.9 x P) x 10^5^ = nmol/h/mg (ARP5R = Sample with Ribose 5-phosphate; ARblank = Sample without Ribose 5-phosphate; P = Sample used (in mg).

### In vitro synthesis of *LvTKT*, *LvPRPS*, *LvIMPDH*, *LvCAD*, *LvDHODH*, *LvUMPS*, *LvCMPK*, and *Luciferase *dsRNAs

dsRNAs were synthesized as described by Su et al. (2014) [13]. Briefly, partial fragments (~ 500–600 bp) of *TKT*, *PRPS*, *IMPDH*, *CAD*, *DHODH*, *UMPS*, *CMPK* and the luciferase (*Luc*) control were amplified using PCR with the corresponding primer sets: Gene-dsF/Gene-dsR or Gene-qF/Gene-dsR or Gene-dsF/Gene-qR (Table 1). The T7 promoter sequence was then incorporated into the amplicon by PCR using the corresponding primer sets: Gene-qF-T7/Gene-dsR, Gene-qF/Gene-dsR-T7 (Table 1). A T7-anchored amplicon was used to generate ssRNA by using T7 RiboMAX™ Express Large Scale RNA Production System (P1320, Promega). Corresponding ssRNA pairs were then combined to form the dsRNA, purified by phenol/chloroform extraction, quantified by Nano-200 Micro-spectrophotometer (Allsheng Instruments, Taiwan) and verified by agarose gel electrophoresis, after which the final dsRNA products were stored at − 80 °C.

### In vivo gene silencing of *LvTKT*, *LvPRPS*, *LvIMPDH*, *LvCAD*, *LvDHODH*, *LvUMPS*, and *LvCMPK* mediated by dsRNA interference

The specific dsRNA, at a dosage of 1 µg/g shrimp weight, was diluted with 0.22 μm-filtered 1x PBS and injected into shrimp (3–5 g body weight) at 72 h before virus injection. Injection of *Luc* dsRNA or PBS served as controls. At 72 h post dsRNA injection, hemocytes were collected (4 pools with 3 shrimp each) and real-time PCR was used to confirm that the *LvTKT*,* LvPRPS*,* LvIMPDH*,* LvCAD*,* LvDHODH*,* LvUMPS*,* and LvCMPK* genes had been specifically silenced by the respective dsRNAs. The remaining shrimp were subjected to WSSV challenge by injection. At 24 h post virus injection, shrimp hemocyte and pleopod samples were collected (4 pools with 3 shrimp each) and used to measure expression of host genes, viral genes and WSSV genome copy number. Statistical analysis was performed as described below.

### Effect of the inhibitors Salirasib, Torin1, LY294002, and Rapamycin (RAP) on the mRNA expression of key host genes involved in de novo nucleotide synthesis during WSSV infection

To evaluate the involvement of the shrimp Ras-PI3K-Akt-mTOR signaling pathway in de novo nucleotide synthesis, shrimp were given intramuscular injections of 100 µL of the following inhibitors: Salirasib (Ras inhibitor, dissolved in 99% EtOH and diluted with 1x PBS, pH 8.0; 35 µg/g shrimp); LY294002 (PI3K/mTORC1 inhibitor, dissolved in 10% DMSO and diluted with 1x PBS; 0.625 µg/g shrimp); Torin1 (mTORC1/mTORC2 inhibitor, dissolved in DMSO and diluted with PEG solvent; 20 µg/g shrimp); and RAP (mTORC1 inhibitor, dissolved in 99% EtOH and diluted with PEG solvent; 0.02 µg/g shrimp) or their vehicles, 2 h before being challenged with WSSV. Samples were collected at 12 and 24 h post WSSV injection. To determine mRNA expression of target genes, all samples were analyzed by real-time PCR and the resulting data was analyzed as described below.

### Statistical analysis

Raw data were collected from at least three independent replicates in each experiment, as detailed in the corresponding methods section. All data were first subjected to the empirical rule, as previously described [36], to detect and exclude statistical outliers. Differences between groups were then analyzed using Student’s t-test for multiple treatments. Data are presented as mean ± SD, with significance indicated by *p < 0.05 or **p < 0.01.

## Results

### Transcriptomic network analysis suggested that enzymes involved in de novo nucleotide synthesis were critical during WSSV infection

**Fig. 1:**
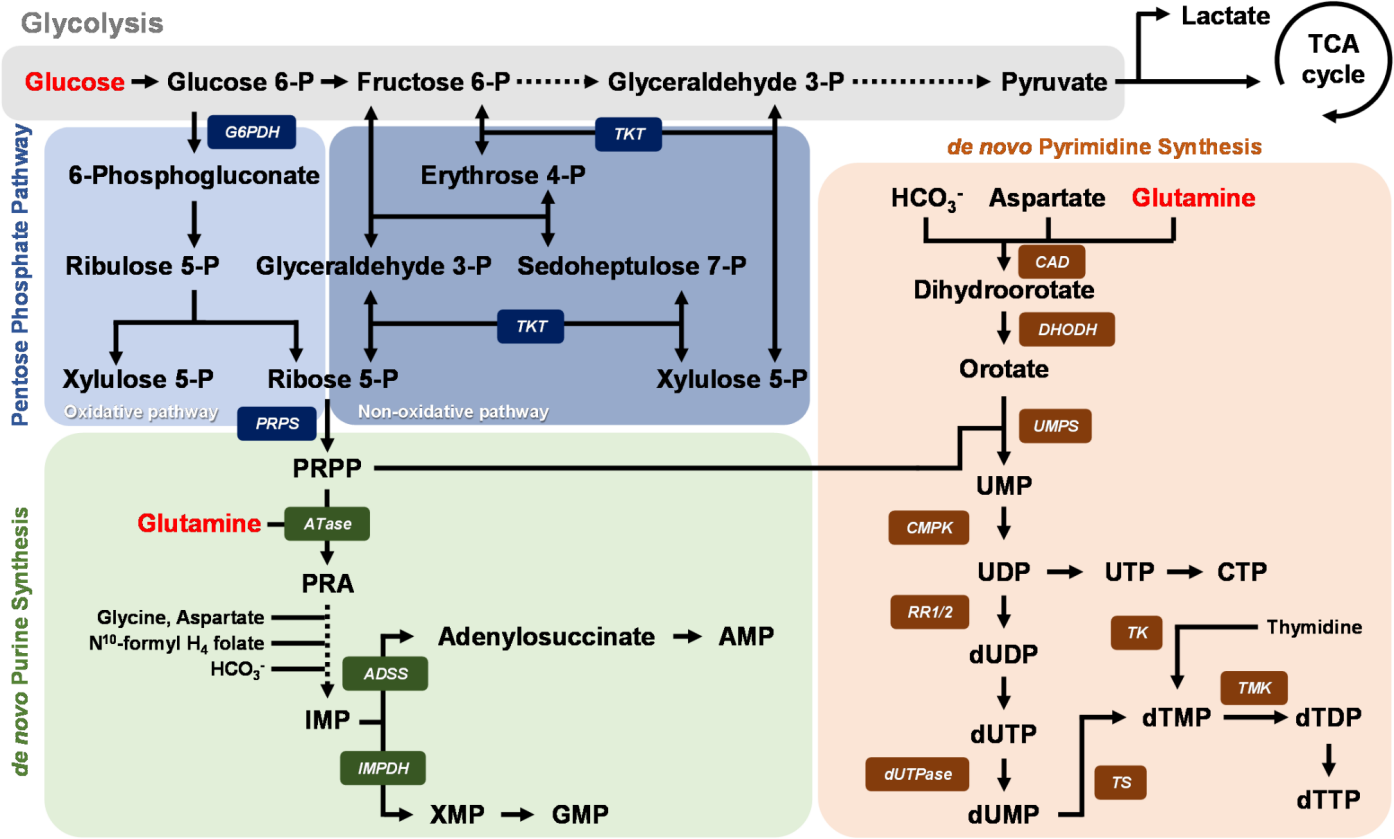
Simplified schematic of de novo nucleotide synthesis. de novo nucleotide synthesis can be divided into the pentose phosphate pathway (blue area), the purine synthesis pathway (green area), and the pyrimidine synthesis pathway (orange area). The pentose sugar backbone of nucleic acids is derived from glucose entering glycolysis (gray area), while glutamine contributes to the synthesis of nitrogenous bases in nucleotides

**Fig. 2:**
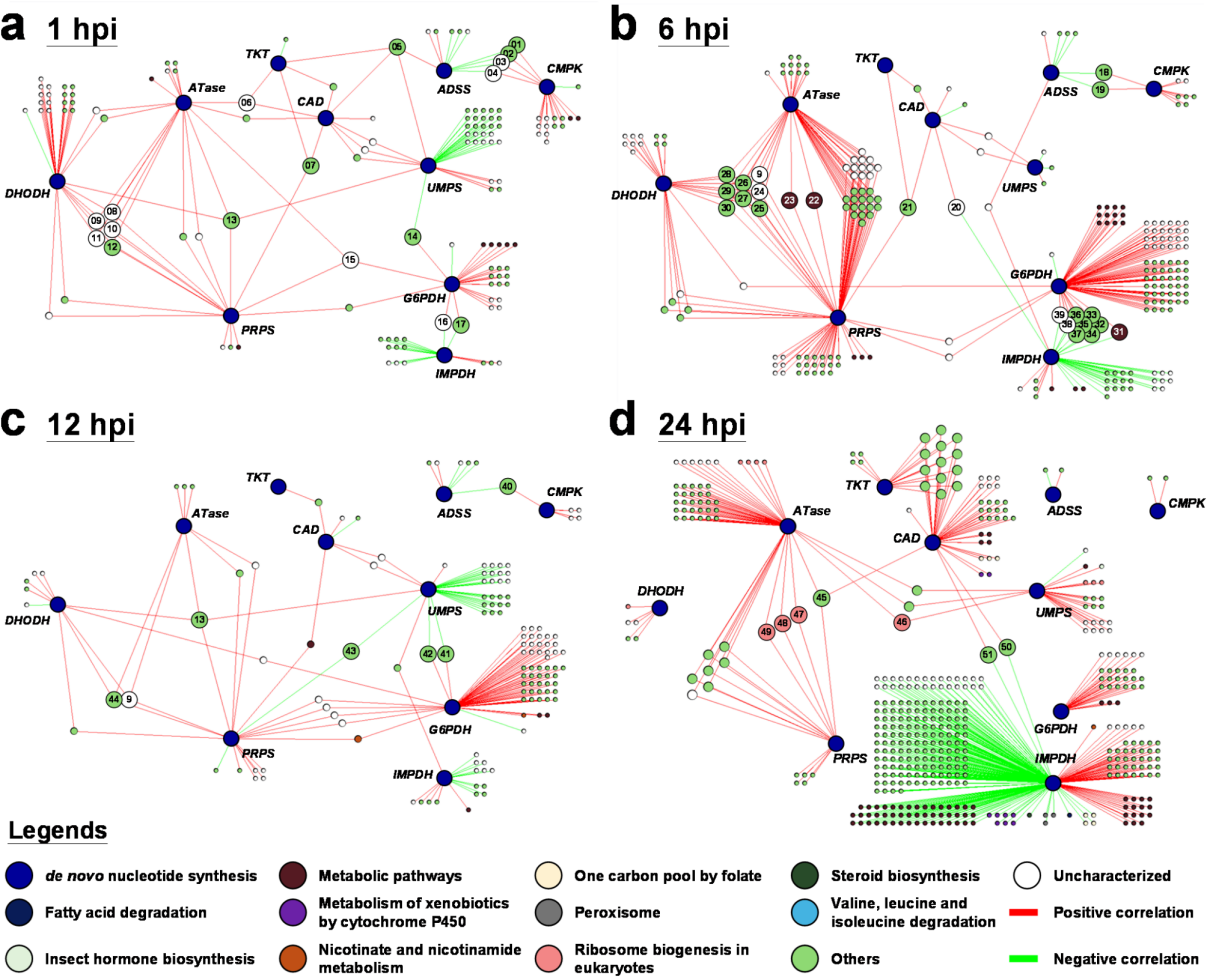
Transcriptomic correlation network for de novo nucleotide synthesis. A correlation network was generated using the criterion of at least a 2-fold change in differential expression between the PBS group versus the WSSV group at (**a**) 1, (**b**) 6, (**c**) 12, and (**d**) 24 h post infection, respectively. A Pearson correlation coefficient threshold as|r| ≥ 0.6. was selected for the transcriptomic network. The contigs are color-coded based on their annotation in the KEGG pathway analysis. Positive and negative correlations between pairs of contigs are indicated by red and green lines, respectively. Further information on the selected highly connected contigs is provided in Supplementary Table 1

To investigate the correlation between WSSV infection and de novo nucleotide synthesis, the *L. vannamei* stomach transcriptomic database in ContigViews was used to conduct a transcriptomic correlation network analysis. For this analysis, host-crucial modulators in the PPP and purine/pyrimidine synthesis pathways (Fig. 1) were selected, and correlation networks were constructed using DEGs that met the criterion of showing at least a 2-fold change between the PBS-treated control group and the WSSV-inoculated group at 1 hpi (extremely early stage of WSSV infection), 6 hpi (early stage of WSSV infection), 12 hpi (WSSV genome replication stage), and 24 hpi (late stage of WSSV infection). In the correlation networks for these four different WSSV infection stages, we found that a number of the host nucleotide metabolism genes were highly associated with WSSV-affected DEGs (Fig. 2). Specifically, these were: *LvG6PDH*, *LvTKT*, and *LvPRPS*, which are involved in the PPP; *LvATase*, *LvADSS*, and *LvIMPDH* which are involved in purine synthesis; and *LvCAD*, *LvDHODH*, *LvUMPS*, and *LvCMPK*, which are involved in pyrimidine synthesis. This is consistent with the hypothesis that de novo nucleotide synthesis plays a critical role during WSSV replication. We also note that *LvIMPDH* exhibited strong negative correlations, suggesting a pivotal role for this enzyme in the metabolic reprogramming that occurs during WSSV infection.

KEGG-based gene annotations were also applied to 820 contigs at the four different infection stages, and the contigs were color-coded accordingly. Although 478 of the contigs (58.3%) showed no significant correlation with any mechanism during enrichment analysis, and 198 other contigs (24.1%) were annotated as uncharacterized, the remaining 144 contigs (17.6%) were significantly associated with functions that included: metabolic pathways, ribosome biogenesis in eukaryotes, metabolism of xenobiotics by cytochrome P450; metabolism of the one-carbon pool by folate; peroxisome metabolism, and nicotinate and nicotinamide metabolism. This suggests a strong association between the modulation of de novo nucleotide synthesis genes and the WSSV-affected DEGs. Based on the positive and negative connections within the network, the highly connected contigs most likely to have a potential influence are listed in Supplementary Table 1.

### Metabolic reprogramming of glucose in WSSV infection favors purine synthesis

**Fig. 3:**
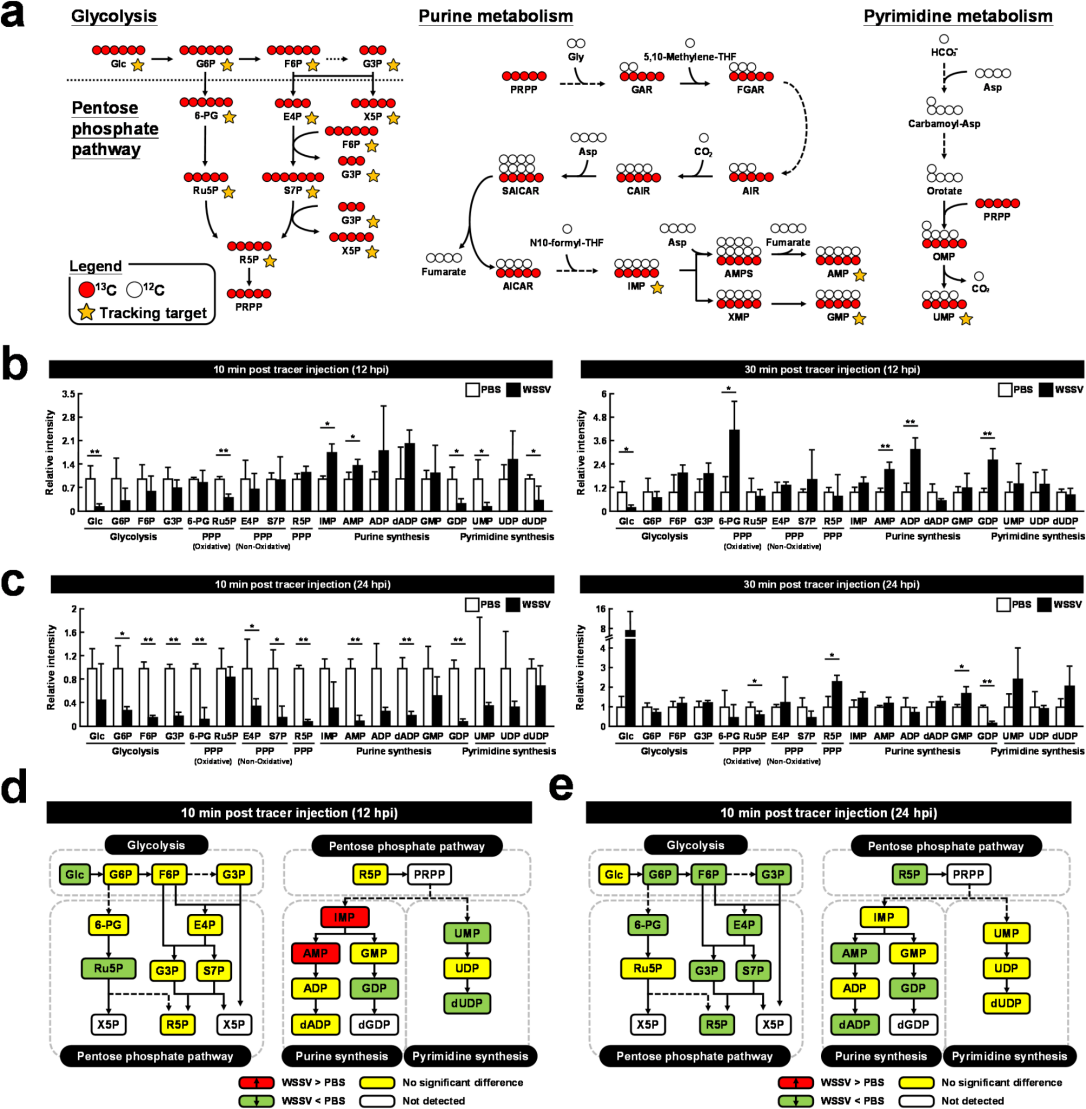
WSSV increased ^13^C-labeled metabolites in the de novo nucleotide synthesis pathway at the WSSV replication stage (12 hpi). (**a**) Schematic indicating how metabolites derived from [U-^13^C]glucose can be traced through the de novo nucleotide synthesis pathway. White circles represent ^12^Carbon, red circles represent ^13^Carbon, and yellow asterisks indicate the targets that were traced. At (**b**) 12 h and (**c**) 24 h post injected with WSSV or PBS, shrimp were injected with [U-^13^C]glucose and hemocytes were collected 10–30 minutes later. Metabolomic analysis of pooled hemocyte samples by LC-ESI-Q-TOF-MS was used to calculate the fold change of each ^13^C metabolite in the WSSV group compared to the corresponding ^13^C metabolite in the PBS group. Each bar represents the mean ± SD. Asterisks indicate differences between the WSSV group and the corresponding PBS control group (* *p* < 0.05, ** *p* < 0.01). Summary of changes in ^13^C-labeled metabolites in the nucleotide synthesis pathway at (**d**) 12 hpi and (**e**) 24 hpi, 10 minutes after [U-^13^C]glucose treatment. Changes in the WSSV group relative to the corresponding PBS control are color-coded as follows: red (significant increase), green (significant decrease), yellow (no significant change), and white (not detected)

To further explore how de novo nucleotide synthesis might be triggered during WSSV infection, stable uniformly labeled [U-^13^C]glucose was used as a metabolic tracer and injected into the abdominal hemal sinus of shrimp during WSSV infection. By measuring the relative intensity of labeled metabolites, we were then able to visualize the impact of WSSV on glycolytic flux and in particular how these changes might contribute to de novo nucleotide synthesis. For this study, shrimp were injected with [U-^13^C]glucose at 12 and 24 h post WSSV challenge, and hemocyte samples harvested 10 and 30 minutes later. The samples were then subjected to metabolomic analysis using LC-ESI-MS. The flow of the ^13^C-labeled carbon through the relevant metabolic pathways is depicted in Fig. 3a.

Compared to the PBS control group, the relative intensities of several [U-^13^C]glucose metabolites in the purine synthesis pathway were significantly increased at 12 h post WSSV injection (Fig. 3b). By contrast, two out of three metabolites in the pyrimidine synthesis pathway showed a significant decrease at the same point (Fig. 3b), suggesting that most of the exogenous glucose might primarily be used for catalyzing and driving purine synthesis rather than pyrimidine synthesis. At 24 h post WSSV injection, most of the ^13^C-labeled metabolites were significantly decreased at 10 minutes after [U-^13^C]glucose injection but recovered at 30 minutes after [U-^13^C]glucose injection (Fig. 3c). Figure 3d-e and Fig. S1 summarizes the measured intensities of the exogenous glucose metabolites at the two different stages of WSSV infection.

### Metabolic reprogramming of glutamine in WSSV infection facilities both purine and pyrimidine synthesis

**Fig. 4:**
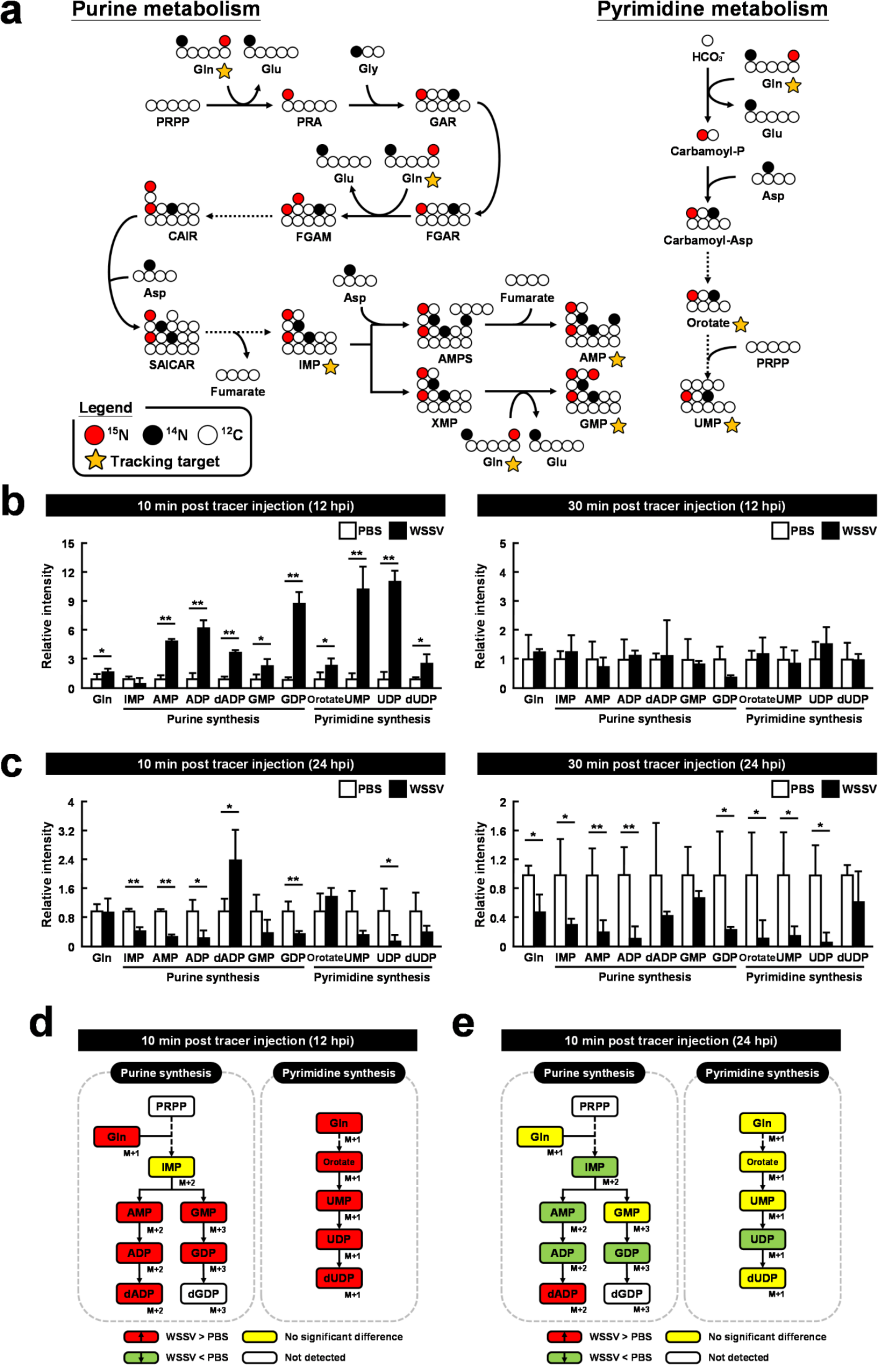
WSSV significantly increased ^15^N-labeled metabolites in the purine/pyrimidine synthesis pathway during the replication stage (12 hpi), but not during the late stage (24 hpi). (**a**) Schematic indicating how metabolites derived from [A-^15^N]glutamine can be traced through the nucleotide synthesis pathway. White circles represent ^12^Carbon, solid black circles represent ^14^Nitrogen, red circles represent ^15^Nitrogen, and yellow asterisks indicate the traced targets. At (**b**) 12 h and (**c**) 24 h post injected with WSSV or PBS, shrimp were injected with [A-^15^N]glutamine and hemocytes were collected 10–30 minutes later. Metabolomic analysis of pooled hemocyte samples by LC-ESI-Q-TOF-MS was used to calculate the fold change of each ^15^N metabolite in the WSSV group compared to the corresponding ^15^N metabolite in the PBS group. Each bar represents the mean ± SD. Asterisks indicate differences between the WSSV group and the corresponding PBS control group (* *p* < 0.05, ** *p* < 0.01). Summary of changes in ^15^N-labeled metabolites in the nucleotide synthesis pathway at (**d**) 12 hpi and (**e**) 24 hpi, 10 minutes after [A-^15^N]glutamine treatment. Changes in the WSSV group relative to the corresponding PBS control are color-coded as follows: red (significant increase), green (significant decrease), yellow (no significant change), and white (not detected)

Since glutamine is a major contributor of the nitrogen that is incorporated into metabolites in the purine and pyrimidine synthesis pathways, the stable isotopically labeled metabolite [A-^15^N]glutamine was used as a metabolic tracer to monitor ^15^N-labeled metabolite concentrations in these pathways during WSSV infection. The flow of ^15^N-labeled nitrogen through the relevant metabolic pathways is depicted in Fig. 4a. Shrimp were injected with [A-^15^N]glutamine at 12 and 24 h post WSSV challenge, and metabolites were extracted from hemocyte samples taken at 10 and 30 minutes after injection.

At 12 hpi and 10 minutes post tracer injection, the relative intensities of ^15^N-labeled metabolites in the purine and pyrimidine synthesis pathways were dramatically higher than in the non-infected control group, with some of the metabolites involved in pyrimidine synthesis showing an increase exceeding 9-fold (Fig. 4b). However, all of the observed metabolites had returned to baseline levels at 30 minutes after injection (Fig. 4b). By contrast, at 24 h post WSSV injection, both the 10- and 30-minute samples showed significant reductions in the levels of most of the ^15^N-labeled metabolites (Fig. 4c). Figure 4d-e and Fig. S2 summarizes the measured intensities of the exogenous glutamine metabolites at the different stages of WSSV infection.

### WSSV infection significantly increased mRNA expression and specific enzyme activity of multiple genes involved in de novo nucleotide synthesis

**Fig. 5:**
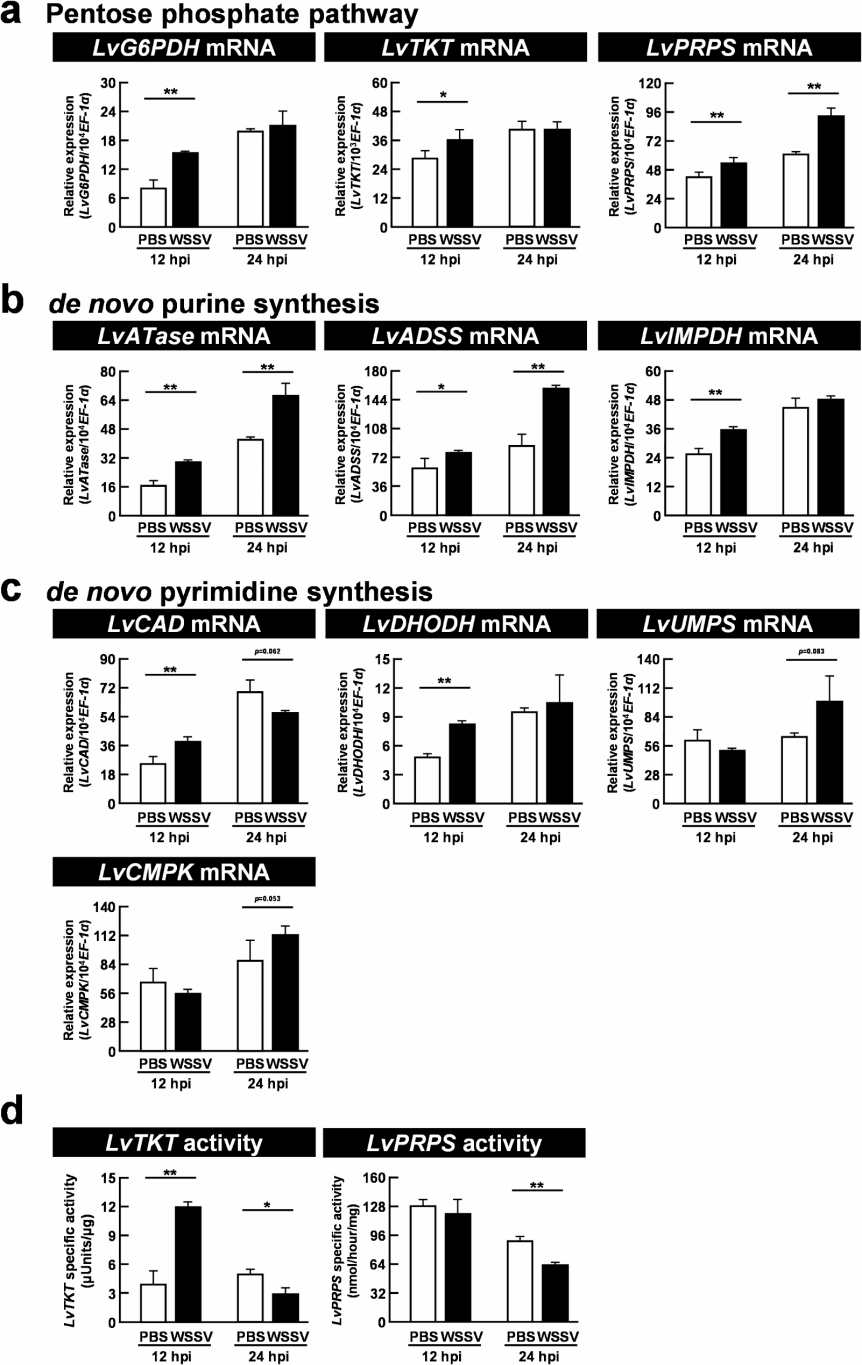
WSSV induced mRNA expression and catalytic activity of multiple genes involved in de novo nucleotide synthesis at the WSSV replication stage (12 hpi). Each bar represents mean ± SD gene expression of (**a**) *LvG6PDH*, *LvTKT*, *LvPRPS*, (**b**) *LvATase*, *LvADSS*, *LvIMPDH*, (**c**) *LvCAD*, *LvDHODH*, *LvUMPS*, and *LvCMPK* in four pooled samples of shrimp hemocytes (3 shrimp/pool) at the WSSV genome replication stage (12 hpi) and late stage (24 hpi). (**d**) Enzyme activity (Mean ± SD) of *LvTKT* and *LvPRPS* in shrimp hemocytes (4 pooled samples; 3 shrimp/pool) at the WSSV genome replication stage (12 hpi) and late stage (24 hpi). Differences between treatment groups are denoted by asterisks (* *p* < 0.05, ** *p* < 0.01)

Since our transcriptomic network analysis and in vivo tracing results both suggested the upregulation of de novo nucleotide synthesis as well as the importance of glucose and glutamine during WSSV infection, we next investigated the expression and activity of ten of the enzymes that drive the de novo nucleotide synthesis pathways (Fig. 5a-c). In the shrimp hemocyte samples, we found that the mRNA expression levels of *LvPRPS*, *LvATase*, and *LvADSS* were significantly upregulated at both the WSSV replication stage (12 hpi) and the late stage (24 hpi). However, *LvG6PDH*, *LvTKT*, *LvIMPDH*, *LvCAD*, and *LvDHODH* were significantly increased only at 12 hpi, while *LvUMPS*, and *LvCMPK* were (slightly) elevated only at 24 hpi. We also found that *LvTKT* protein activity was significantly increased at 12 hpi, whereas both *LvTKT* and *LvPRPS* protein activity were significantly decreased at 24 hpi (Fig. 5d). Taken together, these results suggest that WSSV primarily triggered activation of the PPP and downstream purine/pyrimidine synthesis at the replication stage (12 hpi), which in turn suggests that this activation might be used by the virus to support genome replication.

### In vivo dsRNA-mediated silencing of critical de novo nucleotide synthesis enzymes reduced WSSV gene expression and virus replication

**Fig. 6:**
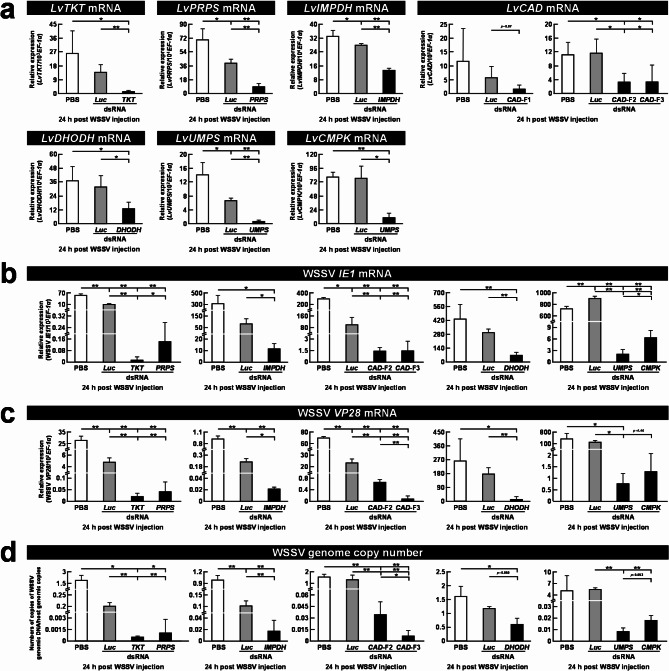
Down-regulation of *LvTKT*, *LvPRPS*, *LvIMPDH*, *LvCAD*, *LvDHODH*, *LvUMPS*, and *LvCMPK* mRNA by specific dsRNA injection and its effect on WSSV *IE1*, *VP28* gene expression and WSSV genome copy number. Gene silencing efficiency at (**a**) 24 h post WSSV injection. WSSV (**b**) *IE1*, (**c**) *VP28* gene expression and (**d**) genome copy number were evaluated after gene silencing of *LvTKT*,* LvPRPS*, *LvIMPDH*, *LvCAD*, *LvDHODH*, *LvUMPS*, and *LvCMPK*. Groups treated with PBS only or with non-specific luciferase (*Luc*) dsRNA were used as control groups. Each bar represents the mean ± SD of mRNA expression. Asterisks indicate differences between the WSSV group and the corresponding control group (* *p* < 0.05, ** *p* < 0.01)

To further investigate the importance of de novo nucleotide synthesis for WSSV replication, dsRNA-mediated gene silencing was used to suppress mRNA expression of critical genes in the PPP and purine/pyrimidine synthesis pathways. To assess the silencing efficiency of the specific dsRNAs, shrimp hemocyte samples were harvested at both 72 h post dsRNA injection (Fig. S3 ) and 24 h post WSSV injection (Fig. 6a). As shown in the figure, the expression of genes involved in de novo nucleotide synthesis were significantly decreased at both time points, confirming gene knockdown. Note however, that when we initially targeted the 5’ upstream region (carbamoyl-phosphate synthetase, *CPS*) of the trifunctional enzyme *LvCAD* (designated as *LvCAD-*F1), the gene expression of *LvCAD* remained unchanged compared to the PBS and *Luc* dsRNA controls. We therefore subsequently targeted the downstream regions of *CPS* (designated as *LvCAD-*F2) and Aspartate Transcarbamylase (*ATC*) (designated as *LvCAD-*F3), which successfully reduced *LvCAD* mRNA expression at both time points.

At 24 h post WSSV injection, the expression of the viral genes *IE1* (Fig. 6b) and *VP28* (Fig. 6c) was significantly suppressed in the silenced groups compared to the PBS and *Luc* dsRNA controls. Viral genome copy numbers were also significantly suppressed in the silenced groups (Fig. 6d). These results provide more evidence that de novo nucleotide synthesis plays a crucial role in WSSV replication.

### *Ras*, *PI3K*, and *mTORC1/C2* are involved in the regulation of de novo nucleotide synthesis

**Fig. 7:**
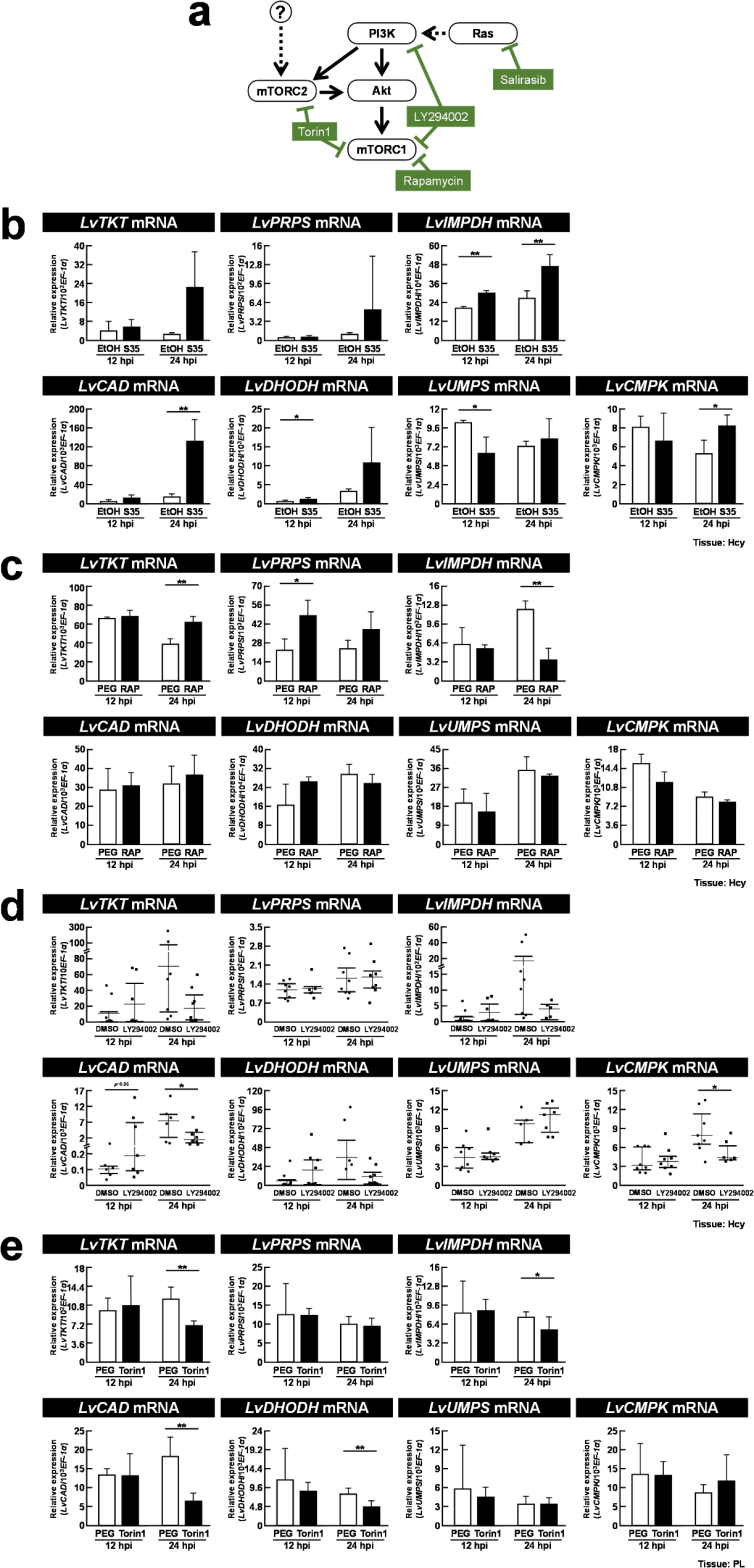
Ras, PI3K, and mTORC1/2 are involved in the regulation of de novo nucleotide synthesis. (**a**) Schematic of the Ras and PI3K-Akt-mTOR pathway and the 4 inhibitors used (green boxes). Target gene expression analysis of shrimp pretreated with: (**b**) Salirasib (S35, 35 µg/g shrimp); (**c**) Rapamycin (RAP, 0.02 µg/g shrimp), (**d**) LY294002 (0.625 µg/g shrimp); and (**e**) Torin1 (20 µg/g shrimp) with samples collected at 12 and 24 h post WSSV injection. The data are presented as mean ± SD, with asterisks indicating significant differences between the inhibitor-treated groups and their corresponding vehicle groups (* *p* < 0.05, ** *p* < 0.01). Hcy: hemocyte; PL: pleopod

While it has previously been shown that the Ras-PI3K-Akt-mTOR signaling pathway is involved in WSSV-induced host metabolic reprogramming [13, 15, 17, 36], here we further investigated whether this same pathway also modulated de novo nucleotide synthesis during WSSV infection. For this study, after treatment with a series of inhibitors (Fig. 7a) followed by WSSV challenge, shrimp hemocyte samples were collected at 12 and 24 h post WSSV injection and the expression levels of genes involved in de novo nucleotide synthesis were determined.

In Salirasib-treated shrimp, gene expressions of *LvIMPDH*, *LvCAD*, *LvDHODH*, and *LvCMPK* were increased (Fig. 7b), implying that Ras is a negative modulator of purine and pyrimidine synthesis. However, there was a significant decrease in *LvUMPS* expression and no change in *LvTKT* and *LvPRPS* expression (Fig. 7b), suggesting that although Ras may act as an activator for *LvUMPS*, it has no effect on ribose synthesis during WSSV infection. In Rapamycin-treated shrimp, the suppression of mTORC1 significantly increased *LvTKT* and *LvPRPS* expression but significantly decreased *LvIMPDH* expression (Fig. 7c). By contrast, in LY294002-treated shrimp, it was found that parallel inhibition of PI3K and mTORC1, as opposed to the inhibition of mTORC1 alone, reduced the expression of *LvCAD*, and *LvCMPK*, while the levels of *LvTKT*, *LvPRPS*, and *LvIMPDH* expression no longer showed any significant difference from those of the vehicle control (Fig. 7d). In Torin1-treated shrimp, it was observed that, compared to mTORC1 inhibition alone, parallel inhibition of mTORC1 and mTORC2 reduced the expression of *LvTKT*, *LvPRPS*, *LvIMPDH*, *LvCAD*, and *LvDHODH* (Fig. 7e). These results provide evidence of the significant involvement of Ras, PI3K, and mTORC1/2 in the regulation of de novo nucleotide synthesis during WSSV infection.

## Discussion

Activation of the Warburg effect and metabolic rerouting not only occurs in different types of cancer cells but also in the pathogenesis of vertebrate viruses, where it addresses the requirements for rapid proliferation [39, 40]. Previous studies have shown that WSSV infection also induces a Warburg effect-like pattern, with an increase in metabolites and the activity of enzymes involved in the PPP and nucleotide synthesis [12, 13]. Our present findings confirm the activation of de novo nucleotide synthesis in the course of WSSV replication and pathogenesis (Figs. 2, 3, 4 and 5). Firstly, in the analysis of the transcriptomic correlation network, we found that crucial host modulators in de novo nucleotide synthesis exhibit a progressively increasing number of correlations, suggesting a strong relationship between these modulators and the metabolic rerouting of the host as the stages of infection progress (Fig. 2). We note however that during the WSSV replication stage and earlier infection stages, some of the key factors were not involved exclusively in nucleotide metabolism. At 1 hpi, *Astakine* (node #12, Fig. 2a) appeared in the network and showed positive correlation with *LvPRPS*, *LvATase*, and *LvDHODH*. *Astakine* is involved in shrimp hematopoiesis [41]. We speculate that WSSV infection increases hemocyte production in immune tissues, requiring a large amount of nucleotides to support cell generation. Consequently, multiple nucleotide metabolism genes are activated. Under this condition, WSSV may hijack the host’s activated metabolic system to facilitate its replication.

Previous studies have reported that at 6 hpi, WSSV promotes the activation of key factors to mitigate the oxidative stress caused by rapid replication [42]. Consistent with this, at 6 hpi, we observed that *S-adenosylmethionine synthase-like* (node #22, Fig. 2b), *glutathione S-transferase class-mu 26 kDa isozyme 47-like* (node #23, Fig. 2b), and *NADPH oxidase 5-like* (node #35, Fig. 2b) appeared in the network and exhibited strong correlations with multiple nucleotide metabolism genes. This suggests that WSSV-induced oxidative stress regulation is activated simultaneously with the PPP, a key route for NADPH generation, and also triggered the downstream purine/pyrimidine synthesis. At this stage, we also found that *Ras-related protein Rac1-like* (node #29, Fig. 2b) exhibited a similar correlation pattern to genes that are up-regulated in response to oxidative stress, suggesting that WSSV-induced oxidative stress regulation may be mediated through *Rac1*. Additionally, *acyl-CoA synthetase short-chain family member 3*,* mitochondrial-like* (node #31, Fig. 2b) and *probable acyl-CoA dehydrogenase fadE25* (node #32, Fig. 2b), which are both related to lipolysis and involved in β-oxidation, also appeared in the 6 hpi network analysis. Although previous studies have reported that WSSV induces host lipolysis at 12 hpi [18], our 6 hpi network analysis suggests that WSSV may have already initiated lipolysis at this stage, potentially supplying energy to support nucleotide synthesis.

In the network analysis at 12 hpi and 24 hpi, although several genes still exhibited correlations with de novo nucleotide synthesis genes, it was unexpectedly difficult to determine their potential roles in metabolism based on known pathways. However, we note that there was a marked negative correlation between *LvIMPDH* and the other DEGs at 24 hpi, suggesting the central importance of *LvIMPDH* in the late stage of WSSV infection. *IMPDH* regulates both nucleotide metabolism and cell proliferation, and it is one of the most important enzymes involved in purine synthesis [43], while other studies have shown that the inhibition of *IMPDH* reduces the replication levels of various vertebrate viruses [44, 45]. In addition to its catalytic activity, *IMPDH* is known to bind to specific single-strand nucleic acid sequences and function as a transcription factor: specifically, by inhibiting histone genes and *E2F*, which is itself a critical transcription factor in the G1/S cell cycle transition [46, 47], *IMPDH* regulates the cell proliferation rate. All of these observations suggest that *IMPDH* might play a pivotal role in WSSV infection, but further investigation is still required.

To support de novo nucleotide synthesis, glucose and glutamine play critical roles as sources of the ribose and nitrogen, respectively, that are needed to form the nucleoside structure. Here, we used labelled [U-^13^C]glucose and [A-^15^N]glutamine, respectively, to confirm their facilitation of de novo nucleotide synthesis and elucidate their contribution to WSSV replication. After [U-^13^C]glucose injection at 12 hpi, we observed an increase in some of the metabolites involved in purine metabolism at both 10 and 30 minutes post injection (Fig. 3b). mRNA expression of genes in these pathways was also elevated at this time point (Fig. 5b). These results reflect how the glycolytic flux contributes to purine metabolism, with increased expression of the relevant genes supporting this process. At 24 hpi, nearly all metabolites in the WSSV-infected group exhibited a sharp decline at 10 minutes after injection. These metabolites then returned to normal levels after 30 minutes (Fig. 3c). We also observed a significant reduction in the activity of two key enzymes during this phase (Fig. 5d). Taken together, these results suggest that by 24 hpi, WSSV had likely completed most of its replication requirements and was transitioning towards the release of viral progeny into the extracellular environment. Meanwhile, in the [A-^15^N]glutamine experiment, we detected a substantial increase in labeled metabolites in both purine and pyrimidine metabolism 10 minutes after injection at 12 hpi (Fig. 4b), indicating that exogenous glutamine was heavily utilized and that it contributed significantly to nucleotide synthesis. Similarly, as with the [U-^13^C]glucose treatment, most metabolites decreased at the late stage of infection (24 hpi, Fig. 4c), during which virion morphogenesis occurs. The results shown in Figs. 3 and 4 further suggest that glutamine’s contribution to nucleotide metabolism may be more significant than that of glucose, and we speculate that this might be due to some of the glycolytic flux being directed toward other pathways [14]. In K-RAS-activated oncogenic NIH3T3 mouse fibroblasts, the crucial contribution of glutamine-derived DNA precursors to cancer cell progression was demonstrated by showing that glutamine deprivation prevented cells from entering the S-phase and significantly reduced cell proliferation, and that these effects were canceled by restoring the four deoxyribonucleotides [48]. However, under the same conditions, the absence of glucose led to increased cell death rather than restricted proliferation [49]. Together, these results suggest that these two important inputs may have different roles in cell survival and proliferation, while emphasizing the importance of glutamine to WSSV replication.

To evaluate the importance of de novo nucleotide synthesis in WSSV replication at the transcriptomic level, we next conducted in vivo animal experiments in which shrimp were challenged with WSSV and the expression of specific genes was assessed (Fig. 5). After WSSV infection, the up-regulation of multiple genes (Fig. 5) provided further confirmation of the WSSV-induced activation of the PPP, purine, and pyrimidine synthesis pathways, while dsRNA silencing experiments indicated the importance of de novo nucleotide synthesis by showing that prevention of this process impeded the successful replication of the virus (Fig. 6).

In the final part of this study, we investigated the mechanisms by which WSSV mediates the rerouting of nucleotide metabolism. Modulation of the Ras-PI3K-Akt-mTOR signaling pathway, a crucial pathway involved in various cellular processes, including glycolysis, PPP, NADPH metabolism, and nucleotide synthesis, has been demonstrated in both viral infections and tumor progression [22, 50–52]. Multiple reports have also indicated its importance in the WSSV infection process [13, 15–17, 36], and one of our previous studies [13] demonstrated that suppression of the PI3K-Akt-mTOR pathway impaired virus replication in terms of viral gene expression and viral genome copies. In this study, at 12 hpi Ras inhibition elevated *LvIMPDH* and *LvDHODH* expression while reducing the expression of *LvUMPS* (Fig. 7b), suggesting that Ras may not exert a consistent regulatory effect on de novo nucleotide synthesis genes at this stage. Similarly, at 24 hpi, there was only weak evidence that Ras might be playing a key role at the late stage of infection, since Ras inhibition significantly increased expression only in three of the genes involved in purine and pyrimidine synthesis (Fig. 7b). When only mTORC1 was inhibited, the expression of pyrimidine-related genes remained unchanged (Fig. 7c). However, at 24 hpi the expression of *LvTKT*, a key enzyme in the PPP, was upregulated, suggesting that mTORC1 may suppress the PPP at the late stage to reduce NADPH production. A reduction in NADPH would subsequently increase ROS levels and potentially facilitate the release of viral progeny from the host cell via cell lysis [12]. Meanwhile, when both mTORC1 and mTORC2 were suppressed, the expression of *LvTKT* significantly decreased, suggesting that mTORC2 may act as an activator of *LvTKT* and is balanced by reciprocal regulation with mTORC1. Additionally, mTORC2 may also be involved in the activation of *LvIMPDH*, *LvCAD*, and *LvDHODH* expression. Based on the current results, we are unable to infer any clear regulatory effect that the Ras-PI3K-Akt-mTOR signaling pathway might have on de novo nucleotide metabolism during WSSV infection. Additionally, these results suggest that additional signaling pathways, or even crosstalk among them, may be involved in regulating WSSV-induced de novo nucleotide metabolism, and we note that several studies have demonstrated multiple signaling pathways that play a key role in WSSV-induced host reprogramming. Using dsRNA silencing, Yang et al. (2020) showed that suppression of the JAK/STAT signaling pathway reduced glycolysis and lipogenesis [53]. Similarly, silencing the transcription factor *HIF1-α* blocked the upregulation of glycolytic genes in shrimp [54]. However, further investigation is needed to determine whether these pathways are also involved in WSSV-regulated de novo nucleotide metabolism.

This study has several limitations that should be acknowledged. Nucleotide metabolism is a highly complex and interconnected network involving multiple metabolic pathways. While this research focused on the major de novo nucleotide synthesis pathways, other routes, such as the salvage pathway and the contributions of specific amino acids, were not yet explored. Additionally, the lack of established shrimp cell lines restricted the ability to perform validations in a controlled cellular environment. As a result, all experiments and validations were conducted using in vivo animal models, which may introduce biological variability and limit the resolution of certain mechanistic insights. Addressing these gaps in future studies could provide a more comprehensive understanding of the metabolic interactions between WSSV and its host.

## Conclusions

Many studies have demonstrated how viruses can hijack the host’s metabolic system during infection to produce essential metabolites required for viral replication. However, the interplay between invertebrates and their viruses, such as shrimp and WSSV, remains underexplored, particularly in the context of nucleotide metabolism. The roles of glucose and glutamine in WSSV infection, specifically within invertebrate hosts, are not yet fully understood. In this study, we employed a novel multi-omics approach combined with in vivo animal experiments to investigate these metabolic interactions. This comprehensive strategy allowed us to uncover critical insights into the reprogramming of shrimp nucleotide metabolism during WSSV infection and its implications for viral pathogenesis. In this study, we reveal that: (1) WSSV infection significantly activates the metabolic flux of de novo nucleotide synthesis pathways; (2) Glutamine, rather than glucose, is preferentially utilized to drive purine and pyrimidine synthesis; (3) WSSV enhances both the expression and activity of key enzymes involved in de novo nucleotide metabolism; (4) de novo nucleotide synthesis is critical for WSSV replication; and (5) The Ras-PI3K-AKT-mTOR signaling pathway is not the primary regulatory mechanism for this activation, suggesting the involvement of alternative pathways. These findings highlight the pivotal role of host nucleotide metabolism in WSSV pathogenesis and suggest potential metabolic targets for controlling WSSV outbreaks in shrimp aquaculture.

## Electronic supplementary material

Below is the link to the electronic supplementary material.

Supplementary Material 1

Supplementary Material 2

Supplementary Material 3

## Data Availability

The datasets used and/or analyzed during the current study are available from the corresponding author on reasonable request.

## Abbreviations

ADSS: Adenylosuccinate synthetase
ATase: Amidophosphoribosyltransferase
ATCase: Aspartate transcarbamylase
CAD: Carbamoyl-phosphate synthetase 2, aspartate transcarbamylase, and dihydroorotase
CMPK: Cytidine/uridine monophosphate kinase
CPSase: Carbamoyl-phosphate synthetase
DEG: Differentially expressed gene
DHODH: Dihydroorotate dehydrogenase
dsRNA: Double stranded RNA
EDC: 1-Ethyl-3-(3-dimethylaminopropyl)-carbodiimide
EF-1α: Elongation factor 1 alpha
G6PDH: Glucose-6-phosphate dehydrogenase
Hpi: Hours post injection
IMPDH: Inosine monophosphate dehydrogenase
KEGG: Kyoto Encyclopedia of Genes and Genomes
*Lv*: *Litopenaeus vannamei*
LC-ESI-MS: Liquid chromatography electrospray ionization mass spectrometry
Luc: Luciferase
PBS: Phosphate buffer saline
PPP: Pentose phosphate pathway
PRPS: Phosphoribosyl pyrophosphate synthetase
RAP: Rapamycin
RR: Ribonucleotide reductase
SPF: Specific pathogen free
TK: Thymidine kinase
TKT: Transketolase
TMK: Thymidylate kinase
TS: Thymidylate synthase
UMPS: Uridine monophosphate synthase
UPLC: Ultra-performance liquid chromatography
WSD: White spot disease
WSSV: White spot syndrome virus
