# Supplementary material for: Glucose- and glutamine-driven de novo nucleotide synthesis facilitates WSSV replication in shrimp
Source: Cell Commun Signal. 2025 Apr 22;23:191. doi: 10.1186/s12964-025-02186-z (PMC12012963; doi:10.1186/s12964-025-02186-z)
Supplement: Supplementary file 1 — Supplementary Material 1 [file 12964_2025_2186_MOESM1_ESM.pdf]

Statement on the Use of Gels and Blots Images in the Study

It is clarified that no data derived from gels or blots images are included in this study. All data presented in this study were obtained through other experimental methods, which are described in detail in the Materials and Methods section of the study.
