# Supplementary material for: Glucose- and glutamine-driven de novo nucleotide synthesis facilitates WSSV replication in shrimp
Source: Cell Commun Signal. 2025 Apr 22;23:191. doi: 10.1186/s12964-025-02186-z (PMC12012963; doi:10.1186/s12964-025-02186-z)
Supplement: Supplementary file 2 — Supplementary Material 2 [file 12964_2025_2186_MOESM2_ESM.docx]

**Supplementary figure 1. Summary of changes in ^13^C-labeled metabolites in the nucleotide synthesis pathway at (a) 12 hpi and (b) 24 hpi, 30 minutes after [U-^13^C]glucose treatment.** Changes in the WSSV group relative to the corresponding PBS control are color-coded as follows: red (significant increase), green (significant decrease), yellow (no significant change), and white (not detected).

**Supplementary figure 2. Summary of changes in ^15^N-labeled metabolites in the nucleotide synthesis pathway at (d) 12 hpi and (e) 24 hpi, 30 minutes after [A-^15^N]glutamine treatment.** Changes in the WSSV group relative to the corresponding PBS control are color-coded as follows: red (significant increase), green (significant decrease), yellow (no significant change), and white (not detected).

**Supplementary figure 3. Gene expression of *LvTKT*, *LvPRPS*, *LvIMPDH*, *LvCAD*, *LvDHODH*, *LvUMPS*, and *LvCMPK* at 72 h post corresponding dsRNA injection.** Groups treated with PBS only or with non-specific luciferase (*Luc*) dsRNA were used as control groups. Each bar represents the mean ± SD of mRNA expression. Asterisks indicate differences between the WSSV group and the corresponding control group (* *p*<0.05, ** *p*<0.01).
